# Anti-platelet drugs and their necessary interaction with endothelial mediators and platelet cyclic nucleotides for therapeutic efficacy^☆^

**DOI:** 10.1016/j.pharmthera.2018.08.004

**Published:** 2019-01

**Authors:** Rebecca B. Knowles, Timothy D. Warner

**Affiliations:** The Blizard Institute, Barts and the London School of Medicine and Dentistry, Queen Mary University of London, London, UK

**Keywords:** Platelet, Endothelium, Dual antiplatelet therapy, Thrombosis, Cyclic nucleotide, AC, Adenylyl cyclase, ACS, Acute coronary syndrome, ADP, Adenosine diphosphate, COX, Cyclooxygenase, DAPT, Dual antiplatelet therapy, GC, Guanylyl cyclase, MACE, Major adverse cardiovascular events, NO, Nitric oxide, NOS, NO synthase, NSAIDs, Non-steroidal anti-inflammatory drugs, PDE, Phosphodiesterase, PFT, Platelet function test, PGI_2_, Prostacyclin, TXA_2_, Thromboxane A_2_

## Abstract

For many millions of patients at secondary risk of coronary thrombosis pharmaceutical protection is supplied by dual anti-platelet therapy. Despite substantial therapeutic developments over the last decade recurrent thrombotic events occur, highlighting the need for further optimisation of therapies. Importantly, but often ignored, anti-platelet drugs interact with cyclic nucleotide systems in platelets and these are the same systems that mediate key endogenous pathways of platelet regulation, notably those dependent upon the vascular endothelium. The aim of this review is to highlight interactions between the anti-platelet drugs, aspirin and P2Y_12_ receptor antagonists and endogenous pathways of platelet regulation at the level of cyclic nucleotides. These considerations are key to concepts such as anti-platelet drug resistance and individualized anti-platelet therapy which cannot be understood by study of platelets in isolation from the circulatory environment. We also explore novel and emerging therapies that focus on preserving haemostasis and how the concepts outlined in this review could be exploited therapeutically to improve anti-thrombotic efficacy whilst reducing bleeding risk.

## 1 Introduction

For many millions of patients at secondary risk of coronary thrombosis pharmaceutical protection is supplied by dual anti-platelet therapy (DAPT). Despite substantial therapeutic developments over the last decade recurrent thrombotic events occur, highlighting the need for further optimisation of therapies, especially in patients at elevated risk of major adverse cardiovascular events (MACE). Notably, but not often emphasized in reviews, anti-platelet drugs interact with cyclic nucleotide systems in platelets and these are the same systems that mediate key endogenous pathways of platelet regulation. The aim of this review is to highlight interactions between the anti-platelet drugs, aspirin and P2Y_12_ receptor antagonists and endogenous pathways of platelet regulation at the level of cyclic nucleotides whilst underlining the importance of the endothelium. We discuss the relevance to concepts such as anti-platelet drug resistance, individualized anti-platelet therapy and the combination of aspirin with other anti-platelet drugs and explore novel and emerging therapies that focus on preserving haemostasis and how the concepts outlined in this review could be exploited therapeutically to potentiate anti-thrombotic potential whilst reducing bleeding risk.

## 2 The endothelium, platelets and haemostasis

The circulatory system has developed a range of haemostatic processes to maintain its integrity. Two principal players in arterial haemostasis are blood platelets and endothelial cells lining the interior surface of all blood vessels. Platelets respond to breakages in the arterial wall by sensing underlying exposed proteins, adhering, activating and attracting in more platelets to rapidly build a platelet plug. This process activates the clotting cascade to add insoluble fibrin strands to strengthen and bind the growing thrombus. In parallel, this cascade of factors is limited by generation of anti-clotting processes that limit clot growth ensuring it remains focused on the local bleeding event.

Since Furchgott and Zawadski's seminal report in 1980 of the obligatory nature of the endothelium in producing blood vessel relaxation (Furchgott & Zawadzki, 1980) there has been a vast quantity of literature published in the area of blood vessel regulation by endothelial cells, building on studies from the 1960s and 1970s by researchers such as Florey, Jaffe, Gimbrone and Vane. These studies characterise endothelial cells as a remarkably diverse cell population in both structure and function with differing properties in different vascular beds, in arterial, venous and microcirculations, and even within the same blood vessel (Aird, 2012). In addition to controlling vasomotor tone the endothelium is vital for haemostatic balance and its phenotypic heterogeneity contributes to how systemic imbalance will affect haemostasis differently between sites to lead to site specific thrombosis (Aird, 2005).

Crucially, endothelial cells regulate haemostasis by reducing the excitability of platelets through production of nitric oxide (NO) and prostaglandin I_2_ (PGI_2_), which provide a constant inhibitory effect upon platelets within the circulation. NO directly stimulates guanylyl cyclase (GC) in platelets to cause the production of cGMP while PGI_2_ acts on IP receptors to stimulate adenylyl cyclase (AC) to produce cAMP. Elevation of either cGMP or cAMP in platelets causes a reduction in platelet reactivity, and the two together are strongly synergistic, as established more than 25 years ago (Radomski, Palmer, & Moncada, 1987). So platelets with elevated levels of cAMP and cGMP are rendered rather unresponsive (Schwarz, Walter, & Eigenthaler, 2001). However, platelets are also equipped with a range of enzymes that rapidly remove cAMP and cGMP, the phosphodiesterases (PDE) (Rondina & Weyrich, 2012). So, a dynamic balance exists; endothelial cell inhibitory mediators will constantly stimulate the formation of cAMP and cGMP and intraplatelet systems will constantly remove them, with the reactive state of platelets determined as a product of these two systems.

To appreciate the importance of these systems in atherothrombosis, it is important to consider where these interactions between platelets and the endothelium take place. Traditionally, text books and review papers discussing atherothrombosis display pictures of large blood vessels consisting of endothelial cells layered on smooth muscle cells, with platelets passing by, and arrows indicating release of NO and PGI_2_ from endothelial cells into the blood and we therefore, envisage that this is where these mediators exert their principal effects on platelets. Indeed, in many areas of cardiovascular research, attention is paid to the larger vessels: the coronary and carotid arteries for instance, because of their associations with acute coronary and cerebrovascular events and so it seems that there is particular relevance of endothelium in these areas (which actually represent infinitesimally small areas when the vast endothelium is contemplated in its entirety) to human disease. When we consider platelet and endothelial cell interactions in this context we visualise large numbers of platelets present for each endothelial cell and so this is how we perceive these interactions throughout the body. Of course, upon plaque rupture the myriad of activating platelets recruited to drive thrombus formation will certainly outnumber local endothelial cells in order to override physiological inhibitory mechanisms. Whilst it is apparently attractive to consider these interactions during the acute event, it makes little sense to focus upon the roles of locally produced inhibitory endothelial cell mediators in the acute process. Rather, it is important to consider outside of this, in homeostasis what kind of relationship between endothelial cells and platelets could actually be exerted in these large vessels?

When contemplating these interactions, one must reflect a moment on the cardiovascular system. In adults, the total blood volume (5 l) contains approximately 1.25 trillion blood platelets but crucially, these are outnumbered by approximately 60 trillion endothelial cells forming an almost 1 kg organ (Aird, 2005). So, in an individual there are many more endothelial cells than platelets, around 50-fold more. The diameter of a platelet is 2-3 μm, the diameter of a capillary 5-10 μm, and the diameter of the proximal LAD around 2.8–4.2 mm (3000–4000 μm). If one considers the volume to internal surface area ratio of a capillary of 8 μm diameter and 1 cm in length it is 0.5; the volume to internal surface area ratio of the proximal LAD of 4 mm diameter and 1 cm length is 1000. So, in capillaries, as compared to large arterial vessels, there is actually a 2000-fold greater ratio of endothelial cells to platelets. In addition, blood flows around 500 times faster in arteries than in capillaries; the blood flow in a healthy coronary artery is 10 to over 100 cm/s, in a capillary 0.1 cm/s. Lastly, while the cross-sectional area of the aorta is 3–5 cm^2^ that of the body's total capillary bed is approximately 4500–6000 cm^2^, comprising the vast majority of the total surface area of the circulation (Tortora & Derrickson, 2011).

Equipped with these principles we can deduce that the principal influence of the 50-fold excess of endothelial cells over platelets must be exerted in the capillaries and not in the large conduit vessels. In the capillaries there is the time and space for the interaction to take place. Platelets will, therefore, leave the capillary beds with elevated cyclic nucleotide tone from an intimate interaction with local endothelial cells that cannot be matched in larger vessels. As at rest normal human cardiac output matches the blood volume, at around 5 l/min, individual platelets, are exposed to the pulmonary and systemic capillary beds every minute. The pulmonary circulation contains multiple isoforms of NO synthase (NOS) and pulmonary endothelial cells are active producers of NO (Gomberg-Maitland et al., 2013). Similarly, it was noted over 30 years ago that the lung is a major producer of PGI_2_ and that this can inhibit platelet reactivity (Hensby, Barnes, Dollery, & Dargie, 1979). Likewise numerous reports show that endothelial cells from vascular beds throughout the body produce NO and PGI_2_ (Zetter, 1981), with the renal vasculature having a large capacity to generate both NO and PGI_2_ (Nasrallah & Hebert, 2005). Of note, mice deficient in PGI_2_ receptors are predisposed to cardiovascular disease and platelets from patients lacking IP receptors have exaggerated responses to arterial damage (Arehart et al., 2008; Cheng et al., 2002). Similarly, humans with dysfunctional GC (Erdmann et al., 2013) or mice lacking eNOS (Ozuyaman et al., 2005) are at elevated risk of thrombosis.

From this we can construct a view of how the endothelium promotes platelet inhibition and blood fluidity; platelets are central to blood clotting and their innate reactivity is balanced by a 50-fold excess of endothelial cells. Taking into consideration human cardiac output, platelets on their journey through the circulation will make intimate contact with endothelial cells in the pulmonary and systemic microvasculature 2–3 times a minute, exposing them to NO and PGI_2_ elevating platelet cGMP and cAMP, which are strongly inhibitory and ‘tame’ platelets through multiple effects including early activation signals such as release of Ca^2+^ from intracellular stores, G-protein activation, and adhesion, granule release and aggregation (Beck et al., 2014; Gambaryan et al., 2004; Schwarz et al., 2001; Smolenski, 2012) including for example, inhibiting GPVI dimerization (Loyau et al., 2012), the inhibition of platelet shape change through regulation of the RhoA-Rho Kinase-MLC phosphatase signalling pathway (Aburima, Walladbegi, Wake, & Naseem, 2017), reducing phosphatidylserine exposure and blunting platelet pro-coagulant activity (Yan, Wang, Yuan, Cheng, & Dai, 2009), blocking thrombin-induced shape change (Jensen, Selheim, Doskeland, Gear, & Holmsen, 2004) as well as regulation of thrombin-induced activation of Rap1b (Benz et al., 2016), regulation of apoptosis (Rukoyatkina, Walter, Friebe, & Gambaryan, 2011) and inhibiting P-selectin expression (Libersan, Rousseau, & Merhi, 2003). Fundamentally, cyclic nucleotides are global inhibitors of platelet function.

**Fig. 1:**
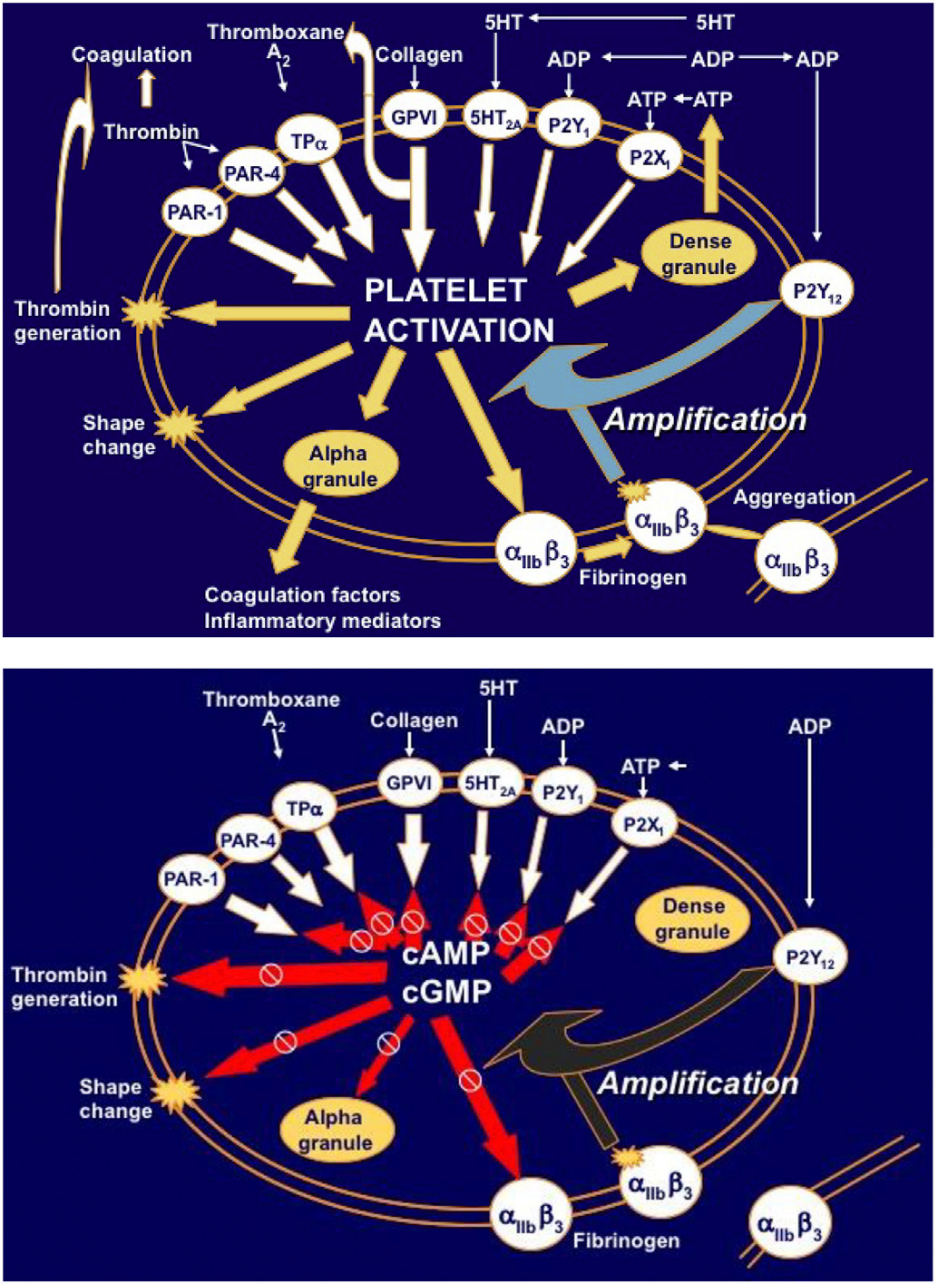
Upper panel (reproduced with permission of Professor RF Storey) summarizes the concept of amplification of platelet activation following stimulation of P2Y_12_ receptors by ADP (Storey, 2006). Strikingly, lower panel demonstrates that targets of P2Y_12_ receptor activation are matched by those under inhibitory regulation by cyclic nucleotides (cAMP and cGMP) (Adams & Feuerstein, 1984; Graber & Hawiger, 1982; Imai, Hattori, Takahashi, & Nozawa, 1983; Lerea, Glomset, & Krebs, 1987; Libersan et al., 2003; Schwarz et al., 2001; Waldmann & Walter, 1989). This is consistent with the concept of amplification being explained by P2Y_12_ receptor activation quenching the inhibitory effects of cyclic nucleotides.

An interesting consideration is how systems have evolved such that fast-flowing platelets can overcome this strong inhibitory tone to aggregate at sites of arterial injury. The answer appears to be that the first arriving platelets release secondary mediators, most notably ADP that activates P2Y_12_ receptors on platelets rapidly leading to blockade of AC, turning off cAMP production, and countering the inhibitory signalling actions of cGMP. Therefore, acting in direct opposition to the powerful, endogenous inhibitory pathways in platelets (Fig. 1). This greatly potentiates platelet activation responses (Cattaneo & Lecchi, 2007; Kirkby et al., 2013; Storey, 2006), a process that is supported by the rapidly acting PDE enzymes that constantly remove cyclic nucleotides (Rondina & Weyrich, 2012; Schwarz et al., 2001). Interestingly, but less well understood P2Y_12_ receptor-dependent PI3K activation could constitute a cGMP-insensitive pathway that supports aggregation in the presence of NO (Kirkby et al., 2013).

Thus, we can draw together many lines of research to propose a single idea of atherothrombus formation with the relationship between the endothelium and platelets at its centre. Endothelial cells produce NO and PGI_2_ that elevate cAMP and cGMP in platelets, and these two systems synergise to produce the greatest inhibition of platelet reactivity. This effect largely takes place within capillary beds, but rapidly moving platelets carry the effects of endothelial cell exposure with them throughout their journey in the vasculature. Exposure of platelets to a damaged blood vessel wall releases ADP that through P2Y_12_ receptor activation pivotally turns off the cAMP and cGMP generating and signalling systems and in concert with activated phosphodiesterases rapidly switches the platelet to being strongly reactive (Fig. 1). These systems are in balance with the time it takes platelets to pass from capillary beds to large vessels. With this concept in mind we can consider the effects and clinical significance of the anti-platelet drugs currently recommended as DAPT in a ‘one size fits all approach’.

## 3 Aspirin

Aspirin, marketed in tablet form since 1899, is established in clinical practice as the default anti-platelet therapy in cardiovascular disease. This is based upon robust data that for “at risk” patients low dose aspirin reduces thrombotic events by around 30% (Patrono, Garcia Rodriguez, Landolfi, & Baigent, 2005). Aspirin acts by irreversibly blocking the cyclooxygenase enzyme (COX) within platelets, inhibiting the production of thromboxane A_2_ (TXA_2_). TXA_2_, when unchecked, drives further aggregation through stimulation of receptors for thromboxane A_2_ (TP receptors) on neighbouring platelets. While aspirin is short-lived in the circulation, it permanently inhibits the COX-1 enzyme through acetylation, and as platelets lack the apparatus to produce replacement COX-1 protein individual platelets remain inhibited for their lifetime. Platelets circulate for around 7–10 days and so the concept has arisen of once a day dosing with aspirin to produce blockade of the entire platelet population. Aspirin is effective in doing this at what is generally referred to today as low dose (75–100 mg/day) which has evolved dramatically from previous cardiovascular doses (900–1500 mg). While this is low dose relative to anti-inflammatory (5 g/day) and doses used for pain (600–650 mg) it is not a low dose for platelets which are completely inhibited (Patrignani, Filabozzi, & Patrono, 1982).

Importantly, in addition to its effects upon platelets, aspirin inhibits COX at other sites in the body (FitzGerald et al., 1983; Warner, Nylander, & Whatling, 2011) with doses of around 600 mg (two standard tablets) producing analgesic and antipyretic effects through inhibiting COX at sites other than the platelet. There is a substantial amount of information regarding the effects of strong, whole body blockade of COX enzymes, and an understanding that this is associated with increased thrombotic risk (Kearney et al., 2006). The generally accepted mechanism for this is changes in the balance of pro-aggregatory TXA_2_ from platelets and anti-aggregatory PGI_2_ from endothelial cells, changes that can be followed by the measurement of urinary metabolites (FitzGerald et al., 1983; Warner et al., 2011) For example, 80 mg aspirin reduced urinary TXA_2_ metabolites by around 80% and PGI_2_ metabolites by around 50%; 325 mg aspirin reduced the metabolites, respectively, by around 95% and 70% (FitzGerald et al., 1983) with the investigators concluding it was unlikely that any dose of aspirin could completely selectively inhibit TXA_2_ synthesis. Others report even stronger effects on PGI_2_ production (Ritter, Cockcroft, Doktor, Beacham, & Barrow, 1989; Warner et al., 2011) and local measures strongly support the notion that aspirin inhibits COX in the vascular endothelium and so reduces PGI_2_ production (Mitchell & Warner, 2006; Warner et al., 2011). One must conclude that even at low, anti-thrombotic doses, aspirin produces substantial inhibition of COX within the vasculature, both in platelets and in endothelial cells. As shown in multiple models, reduction in PGI_2_ signalling in platelets causes increased *in vivo* activation.

## 4 P2Y_12_ receptor blockade and dual anti-platelet therapy

As knowledge regarding anti-thrombotic therapy develops, it has become accepted that administration of blockers of the ADP P2Y_12_ receptor, such as clopidogrel, prasugrel or ticagrelor, together with aspirin further reduces the risk of acute thrombotic events (Wallentin et al., 2009; Wiviott et al., 2007; Yusuf, et al., 2001). Because of the manner in which this therapeutic approach evolved randomised clinical trials conducted over two decades and notably since the introduction of more efficacious P2Y_12_ blockers have always been conducted in the presence of aspirin. Clopidogrel is associated with wide individual variability in pharmacodynamic response so with a third of patients not achieving satisfactory platelet inhibition (Gurbel, Bliden, Hiatt, & O'Connor, 2003), it may well be that for patients receiving clopidogrel addition of aspirin provides further anti-thrombotic protection. However, at their standard clinical doses prasugrel and ticagrelor produce strong and consistent P2Y_12_ blockade (Gurbel et al., 2009). With these agents now established in clinical practice it is an important time to readdress the question of whether patients should also receive aspirin.

We have suggested previously that addition of aspirin to strong P2Y_12_ blockade may produce little additional inhibition of platelet function, when platelets are considered in isolation. This is as several lines of evidence suggest there is not a simple additive effect between the two treatments. P2Y_12_ antagonists inhibit not only ADP-induced platelet aggregation but also TXA_2_ pathways of platelet activation (Armstrong et al., 2011). As outlined above data from studies of NSAIDs indicate that whole body inhibition of COX increases thrombotic risk, and aspirin inhibits COX at sites other than the platelet. Reduction in vascular PGI_2_ production, for instance, can reduce platelet cAMP, increase platelet reactivity and so increase the potential for thrombosis. The incremental increase in platelet inhibition provided by aspirin on top of strong P2Y_12_ receptor blockade may well be insufficient to balance this thrombogenic effect. So, while consideration of platelet activation pathways in isolation could lead to the conclusion that addition of aspirin to strong P2Y_12_ blockade increases platelet inhibition, consideration of platelet activation pathways *in vivo,* within the milieu of the circulation may lead to the conclusion that addition of aspirin actually decreases platelet inhibition. Aspirin also increases bleeding risk, particularly within the gastrointestinal tract, increases blood pressure and promotes fluid retention, providing additional reasons to question the overall clinical benefit of aspirin's addition to potent third generation P2Y_12_ blockers.

## 5 Dual anti-platelet therapy, PGI_2_ and NO

As a further layer of complexity, blockade of platelet P2Y_12_ receptors also significantly increases the sensitivity of platelets to the inhibitory effects of both PGI_2_ (Cattaneo & Lecchi, 2007) and NO (Kirkby et al., 2013). This is the flipside of the observation that P2Y_12_ activation reduces the inhibitory effects of cyclic nucleotide signalling pathways within platelets; *i.e.* P2Y_12_ activation greatly increases platelet excitability. When P2Y_12_ receptors are blocked, releasing AC, the effects of PGI_2_ and NO acting through cAMP and cGMP signalling pathways are unimpeded and so their inhibitory effects are increased relative to P2Y_12_ receptor uninhibited platelets. As PGI_2_ and NO have synergistically inhibitory effects upon platelets, the interaction with P2Y_12_ receptor blockers actually provides a powerful three way synergistic effect: NO, PGI_2_ and P2Y_12_ blockade are inhibitory individually, synergise with each other in individual pairs, and synergise still further as a trio providing potent platelet inhibition (Chan et al., 2016). Understanding this powerful interaction has therapeutic and diagnostic implications for DAPT. It provides even further cause to question the benefit of addition of aspirin to strong P2Y_12_ blockade; inhibition of PGI_2_ production will lessen this three-way synergy and increase platelet reactivity beyond that predicted for loss of PGI_2_ alone (which by itself has been suggested as responsible for the pro-thrombotic effects of NSAIDs). This could be particularly relevant to patients with endothelial dysfunction and already compromised endothelial mediator production. It also has significant implications for platelet function testing (PFT) and individualized therapies. Whilst high on-treatment ADP reactivity is linked to negative outcomes following PCI (Stone et al., 2013), randomised trials have not demonstrated benefit of adjusting anti-platelet therapy based on PFT (Tantry, et al., 2013). Emergence of these interactions implies that endothelial mediator production is an important determinant of P2Y_12_ therapeutic efficacy and endothelial function testing alongside PFT could enhance risk prediction, identifying those who would benefit from escalated P2Y_12_ therapy in a personalized therapeutic manner.

## 6 Implications for high risk patient groups

Alongside patients with traditional cardiovascular risk factors, patients with PAD, CKD and COPD represent high-risk populations for atherothrombosis and have increased mortality despite DAPT (Anavekar et al., 2004; Morillas, et al., 2009; Salisbury, Reid, & Spertus, 2007). Mechanisms are not fully understood but shared risk factors, inflammation, accelerated atherosclerosis, oxidative stress and underuse of recommended therapies are proposed. Whilst usually considered separately, all are in fact associated with capillary bed destruction, significant microvascular endothelial dysfunction and disruption of NO and PGI_2_ synthesis. The negative impact of COPD following ACS has been linked to smoking but interestingly, also to reduced pulmonary function independent of smoking (Friedman, Klatsky, & Siegelaub, 1976). The pulmonary vasculature is affected in emphysema with remodeling culminating in altered pulmonary circulation, loss of expression of PGI_2_ synthase and eNOS and decreased PGI_2_, 6-keto-PGF_1α_, PGIS messenger RNA and protein expression, as well as disturbed L-arginine metabolism, reduced NO bioavailability and increased ADMA compared to healthy lungs (Dinh-Xuan et al., 1991). Both PGI_2_ deficiency and impaired NO pathways are implicated in the pathogenesis of PAD with diffuse vascular damage in different territories and elevated ADMA and SDMA levels predicting worse outcome (Morillas, et al., 2009). Increased ADMA levels, and disturbances in PGI_2_ biology have also long been associated with CKD (Vallance, Leone, Calver, Collier, & Moncada, 1992) (Nasrallah & Hebert, 2005). Endothelial dysfunction of pulmonary, renal and peripheral capillary beds, all important producers of PGI_2_ and NO could lead to imbalances of these endothelial mediators, their specific synthases and receptors, all of which are linked to thrombosis (Reid & Kinsella, 2015). Impaired synergy between NO, PGI_2_ and P2Y_12_ antagonists could reduce the therapeutic potential of these agents. Notably, other high-risk groups (diabetics and hypertensives) also display microvascular pathophysiological changes and elevated MACE. This could partly explain why these patients have poorer outcomes despite DAPT with third generation P2Y_12_ antagonists. Recognizing this opens possibilities for more efficacious treatments.

## 7 Novel and future anti-platelet therapies

As discussed above, DAPT with aspirin and P2Y_12_ receptor antagonists represents the cornerstone of therapy for the treatment of atherothrombosis but 10% of ACS patients still experience recurrent thrombotic events and DAPT also increases the risk of bleeding. Indeed, it is often expressed that a ceiling has been reached with current antiplatelet agents and an increase in potency using these approaches will be offset by an increased bleeding risk (McFadyen, Schaff, & Peter, 2018). Notably, differences between haemostasis and thrombosis have started to emerge, identifying new regulators of thrombus formation as targets that potentially may not interfere with haemostasis. Other recent data indicate reasons why existing anti-platelet therapies may lack efficacy. *In vivo* experiments, have shown a hierarchical structure within developing thrombi identified as being composed of two distinct regions: a haemostatic core composed of closely packed, fully activated platelets representing the primary site of fibrin deposition which is highly dependent on ADP, TXA_2_ and thrombin activity; and an outer shell, the propagating thrombus that, in contrast contains platelets that are loosely packed, in a low-activation state regulated by different mechanisms such as PI3Kβ, PDI activation and αIIbβ3 signalling, potentially making these platelets refractory to current antiplatelet therapies (Stalker et al., 2013). With this improved understanding the ability to modulate thrombosis without affecting haemostasis may be closer at hand. In the following section of this review we consider current pre-clinical and clinical data indicating the future directions of antiplatelet therapies.

The central driver of thrombus formation is thrombin for which reason it is a key target for anti-thrombotic therapies. These are aimed at the processes leading to thrombin activation, at thrombin itself, and at thrombin receptors. Direct factor Xa inhibitors stop the conversion by factor Xa of prothrombin into thrombin. The ATLAS ACS 2-TIMI 51 trial has demonstrated that the addition of the factor Xa inhibitor, rivaroxaban, on top of DAPT leads to significant reductions in cardiovascular death but at the cost of increased bleeding (Mega, et al., 2009). This has resulted in the recommendation that rivaroxaban can be used in ACS patients but should be restricted to patients receiving aspirin and clopidogrel with low bleeding risk (Ibanez et al., 2017). Interestingly, however, the APPRAISE-2 trial assessing the effects of apixaban in addition to DAPT failed to find a reduction in the primary endpoint of cardiovascular death, MI or stroke but did find an increase in TIMI bleeding leading to a premature end to the trial (Alexander et al., 2011). The first direct thrombin inhibitor to be tested in addition to DAPT was dabigatran, which in the RE-DEEM trial demonstrated an increase in major bleeding with no reduction of the primary endpoint of cardiovascular death, MI or stroke meaning dabigatran is not indicated in the treatment of ACS (Oldgren et al., 2011). The only available PAR-1 inhibitor, vorapaxar, received FDA approval in 2014 but its use has been associated with an increase in bleeding (Morrow et al., 2012; Tricoci et al., 2012). To minimize this risk vorapaxar is approved as add-on therapy to aspirin or DAPT including clopidogrel but not DAPT including ticagrelor or prasugrel (Ibanez et al., 2017). However, as DAPT with ticagrelor and prasugrel is becoming standard practice it is difficult to imagine an expanded role for vorapaxar. The role of cangrelor, the first i.v. P2Y_12_ inhibitor in the treatment of ACS after FDA approval in 2015 following the CHAMPION PHEONIX trial, also remains to be established (Bhatt et al., 2009). Parmodulins, PAR-1 antagonists distinct to vorapaxar, are also in development with the hope that they produce less bleeding (Flaumenhaft & De Ceunynck, 2017). These represent worthwhile candidates, as rather than inhibiting all downstream signalling from PAR-1, they selectively inhibit platelet and endothelial cell activation and also spare cryoprotective signalling mechanisms in endothelial cells (Aisiku et al., 2015).

In addition to the well-known targets described so far, notably COX-1, P2Y_12_ receptors and thrombin, recent research has identified a broad scope of platelet therapeutic targets. Inhibitory toxins, antibodies, ligand mimetics, nucleotide-based aptamers and soluble recombinant forms of receptor are in development as means of modulating GPVI mediated adhesion pathways, GPIb-IX-V adhesive function and signalling, αIIbβ3 outside in signalling, phosphatidylinositol 3-kinase-beta (P13Kbeta) and protein disulphide-isomerase activation.

Interactions between GPIb and vWF that occur at sites of vascular injury under conditions of high shear stress, conditions which are found in stenotic arteries, are vital in regulation of thrombus growth. Therefore, the GPIb–IX–V axis has been targeted using a variety of agents including antibodies against GPIb or vWF, anti-vWF aptamers, a GPIb antagonist derived from snake venom and recombinant fragments of GPIb or vWF. Unfortunately, the development of caplacizumab, a humanized single-variable-domain immunoglobulin (nanobody) directed towards vWF that has antithrombotic effects was stopped due to an unfavourable bleeding profile (Bartunek et al., 2013). The aptamer (ARC1779) showed promise in phase II trials reducing cerebral thromboembolism however again use of this compound was associated with unacceptable haemorrhagic complications and its development was halted (Markus et al., 2011).

GP IV is the main collagen platelet receptor, critical for the process of αIIbβ3 activation following platelet exposure to collagen which is rich in atherosclerotic plaques. Supporting the idea of this as a therapeutic target with reduced risk, GPVI deficiency is only associated with a mild bleeding phenotype (Dutting, Bender, & Nieswandt, 2012). The GPVI pathway may be inhibited by compounds which induce depletion of platelet GPVI, or by blocking antibodies, or by using mimics of GPVI which bind to collagen and mask its platelet activating epitopes (Ungerer & Munch, 2013). The first tested anti-GPVI antibodies were associated with acute thrombocytopenia or platelet GPVI depletion so their development was halted. However, revacept is a promising recombinant dimeric form of the ectodomain of GPVI fused to the fragment crystallizable (Fc) region of human immunoglobulin G1 (IgG1) (Ungerer et al., 2011) which has shown benefit in phase I trials and is now being tested in phase II trials. Another approach is through blockade of the collagen-binding site of GPVI with high affinity using the antigen-binding fragment of a mouse monoclonal antibody, known as 9O12.2 which has shown initial antithrombotic potential (Ohlmann et al., 2008).

Also in development are innovative ways of targeting αIIbβ3, activated by ‘inside out’ signalling, this is the most abundant platelet receptor and has several ligands other than fibrinogen and vWF (Reheman et al., 2005). Currently clinically available αIIbβ3 inhibitors (abciximab, epitifibatide and tirofiban) reduce the incidence of MI and death but are associated with significant bleeding. In early stages of development are compounds hoped to produce less bleeding risk, such as RUC-4 that interferes with Mg^2+^binding to αIIbβ3 (Li et al., 2014) and therapies that target only active αIIbβ3, amongst others, scFvSCE5, a urokinase plasminogen activator fused to an antibody that binds selectively to activated αIIbβ3 (Fuentes et al., 2016). Other recently described targets are the endogenous thiol isomerase function of the integrin PSI domain near the N-terminus of the β3 subunit (Zhu et al., 2017) and it has been demonstrated that it is potentially possible to target specific and precise signalling functions of.

αIIbβ3 involving Gα13 and talin associations, which could inhibit thrombus formation as effectively as αIIbβ3 inhibitors but without the bleeding risk (Shen et al., 2013). Interestingly, other members of the integrin family such as α2β1, α5β1 and α6β1 (the main laminin receptor) (Schaff et al., 2013) are also being considered as future antiplatelet targets. Another potential approach is to exploit CD39, an ectonucleoside triphosphate diphosphohydrolase which hydrolyzes ADP, with promising data found in a mouse model employing the administration of a fusion protein of solCD39 and an antibody specific for αIIbβ3 active form (Hohmann et al., 2013).

The protein disulfide isomerase (PDI) family of thiol isomerases have been shown to be important for thrombus formation and are secreted from activated platelets and endothelial cells at sites of vascular injury. Critically, inhibition of PDI blocks platelet thrombus formation and fibrin generation (Furie & Flaumenhaft, 2014). Inhibition of PDI by antibodies or small molecule inhibitors blocks thrombus formation and therefore, unsurprisingly efforts have been made to develop PDI inhibitors (Flaumenhaft, Furie, & Zwicker, 2015). Isoquercetin, a flavonoid quercetin, and ML359 are examples of PDI inhibitors undergoing testing in both venous and atherothrombosis with positive results. Most interestingly, and in keeping with the concepts presented in this review, a group of compounds that activate cGMP production by soluble guanylyl cyclase have been shown to reduce thrombus formation in animal models (Stasch, Pacher, & Evgenov, 2011).

Finally, given the myriad of platelet receptors identified as having roles in thrombus formation and regulating other platelet functions, there are many other targets under consideration including antagonists of CD40/CD40L, P-selectin/PSGL-1, Toll-like receptors and GLP-1R (Cameron-Vendrig et al., 2016), and inhibitors of the signalling molecules Syk (Stegner, Haining, & Nieswandt, 2014) and Btk (Kamel et al., 2015), although again with a risk of increased bleeding. As a summary of these existing and emerging therapies, Table 1 lists current anti-platelet therapies and Table 2 summarizes potential new anti-platelet therapies in development.

**Table 1:** Current anti-platelet therapies.

Class of agent	Drug name
Cyclooxygenase inhibitor	Aspirin Triflusal
P2Y_12_ inhibitors	Clopidogrel (Plavix) Prasugrel (Effient) Ticagrelor Brillanta) Cangrelor (Kengreal)
αIIbβ3 inhibitors	Abciximab (Reopro) Eptifibatide (Integrillin) Tirofiban (Aggrastat)
Phosphodiesterase inhibitors	Dipyridamole (Persantine) Cilostazol (Pletal)
PAR1 antagonist	Vorapaxar (Zontivity)
Direct thrombin inhibitors	Bivalirudin (Angiomax) Dabigatran (Pradaxa)
Direct factor Xa inhibitors	Apixaban (Eliquis) Rivaroxaban (Xarelto Edoxaban (Lixiana)

**Table 2:** Potential new emerging anti-platelet agents in development.

Class of agent	Drug name
GPVI antagonists	Revacept Losartan scFv 9012
α2β1 inhibitor	EMS16
GPIb-IX-VWF axis inhibitor (Anti-vWF) Anti-vWF aptamers Anti-vWF nanobody	ARC1779 (Halted), ARC15105 Caplacizumab (Halted), ALX-0081
GPIb-IX-VWF axis inhibitor (Anti-GPIb-IX)	Anfibatide H6B4 NIT family mAbs
P-selectin inhibitors	rPSGL-Ig PSI-697 PSI-421
CD40 inhibitor	Anti-CD40 Ab
αIIβ3 inhibitors	RUC-4 Anti-PSI Domain mAbs scFvSCE5
TP antagonist	Ifetroban, Terutroban
Parmodulins	RWJ-58259
PDI inhibitors	ML359 Isoquercetin
GLP-1R agonist	Exenatide, Liraglutide, Lixisenatide, Albiglutide, Dulaglutide, Semaglutide
Toll-like receptor antagonists	Ginkgolide B
α5β1	PHSCNK, JSM6427
α6β1	GoH3
PI3Kβ	SAR260301, GSK2636771
CD 39	Targ-CD39
Syk inhibitor	BI1002494
BTK inhibitor	LFM-A13
Direct GC activators	YC-1, BAY 41–2272, BAY 58–2667

Whilst the therapies outlined in this section are of great interest and hold promise in the advancement of the treatment of atherothrombosis, it must be highlighted that some have only been established in animal models, most are associated with increased bleeding and all require translation and further trials before being used as antiplatelet therapies. Despite these ongoing research efforts, at the present time the weight of evidence supports the clinical efficacy of DAPT in atherothrombosis and this remains the standard of care. Given the growing evidence that we have presented in this review we suggest that our current focus should be upon making the best use of approved drugs. In particular, it is timely to evaluate the efficacy of third generation P2Y_12_ inhibitors in the absence of aspirin.

## 8 Future of dual anti-platelet therapy without aspirin

Despite DAPT, MACEs remain at 10% and even higher in at risk patient groups fuelling research and discussions regarding optimal therapy. As part of these discussions, aspirin's role in combination therapies is currently being revisited. A notable example is the WOEST trial that demonstrated removal of aspirin from triple therapy (aspirin plus clopidogrel plus warfarin) reduced bleeding and mortality but also unexpectedly, thrombotic events (Dewilde, et al., 2013). Impetus for discussions into the role of aspirin in DAPT has come from the PLATO trial that indicated a reduced efficacy of ticagrelor in North American patients, associated with a negative interaction with higher doses of aspirin (Wallentin et al., 2009). Whilst TRITON-TIMI didn't reflect these results, lower aspirin doses (75 mg–162 mg) were recommended alongside prasugrel and events generally occurred early in the trial before the impact of changes in vascular resistance could occur (Wiviott et al., 2007). The CURE study however, did show a non-significant 23% MACE increase and increased bleeding with higher doses of aspirin (Yusuf, et al., 2001). The randomised clinical trials, GLOBAL-LEADERS (Vranckx et al., 2016) and TWILIGHT (Baber et al., 2016) investigating the relative benefits and risks of aspirin and ticagrelor both singularly and in combination are expected to provide important insights to inform this debate when they report later this year. If aspirin were proven to provide little additional benefit to strong P2Y_12_ blockade, then drugs such as prasugrel or ticagrelor alone could become standard of care. DAPT could then become third generation P2Y_12_ antagonist plus another drug, perhaps those under development touched upon above. It is interesting to speculate that enhancing inhibitory cyclic nucleotide pathways in platelets could represent an effective therapy. The benefits of adding cilostazol (PDE3 inhibitor) to DAPT to reduce MACE without increasing bleeding have been demonstrated (Lee et al., 2011). The mechanisms underlying the effects are not clear, but potentiation of P2Y_12_ blockade through increased cyclic nucleotides could be contributory. If so, removal of aspirin could further reduce MACE by increasing the levels of PGI_2_. Interestingly, the PDE5 inhibitor, dipyridamole, was not beneficial (Park et al., 2014), in keeping with the synergy between P2Y_12_ blockade, NO and PGI_2_ being cAMP dependent (Chan et al., 2016). Agents aimed at directly increasing platelet cyclic nucleotides (synthetic PGI_2_ analogues or direct GC activators) could represent another treatment option. The use of such agents is generally limited by their relaxant effects upon the blood vessel wall which leads for instance to hypotension and headache. However, through enhancement of their inhibitory potencies on platelets by P2Y_12_ blockade, these agents could be provided at effective anti-thrombotic doses that produce lesser effects on the vasculature. By focusing on promoting endogenous inhibitory mechanisms rather than further inhibiting pro-aggregatory pathways in platelets with a third anti-platelet agent, this newer DAPT approach could diminish platelet excitability to reduce thrombosis without increasing bleeding. The application of these endothelial enhancing therapies to those high-risk patients discussed could represent a step towards personalized therapy and improving patient outcomes that can be used both to optimize existing anti-platelet therapies and to provide a therapeutic basis upon which to add new anti-platelet drugs.
